# *Postexit* Surface Engineering of Retroviral/Lentiviral Vectors

**DOI:** 10.1155/2013/253521

**Published:** 2013-04-17

**Authors:** Christoph Metzner, Feliks Kochan, John A. Dangerfield

**Affiliations:** ^1^Institute of Virology, University of Veterinary Medicine Vienna, Veterinärplatz 1, 1210 Vienna, Austria; ^2^Anovasia Pte Ltd, 20 Biopolis Way, 05-518 Centros, Singapore 138668

## Abstract

Gene delivery vectors based on retroviral or lentiviral particles are considered powerful tools for biomedicine and biotechnology applications. Such vectors require modification at the genomic level in the form of rearrangements to allow introduction of desired genes and regulatory elements (genotypic modification) as well as engineering of the physical virus particle (phenotypic modification) in order to mediate efficient and safe delivery of the genetic information to the target cell nucleus. Phenotypic modifications are typically introduced at the genomic level through genetic manipulation of the virus producing cells. However, this paper focuses on methods which allow modification of viral particle surfaces after they have exited the cell, that is, directly on the viral particles in suspension. These methods fall into three categories: (i) direct covalent chemical modification, (ii) membrane-topic reagents, and (iii) adaptor systems. Current applications of such techniques will be introduced and their advantages and disadvantages will be discussed.

## 1. Introduction: Why Modify Retroviral Surfaces?

**Figure 1:**
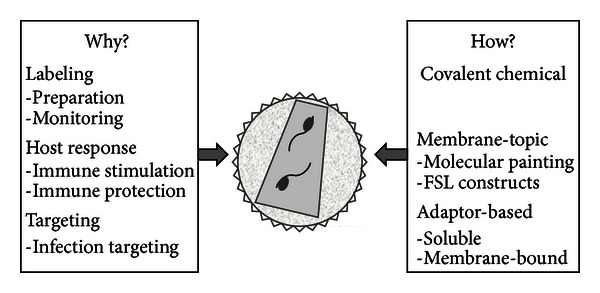
Overview of *postexit* modification of R/LV vector surfaces.

The main task of a viral vector is to deliver its nucleic acid cargo with high efficiency, typically as a means of gene delivery with therapeutic purpose. However, historically there have been drawbacks, especially when considering the very modest successes of *in vivo* delivery. In order to achieve increased efficiency, precision, safety, and comparable ease of preparation and application, a number of special functions may be required from the vector in addition to delivery of genetic material, some of scientific nature and some more related to issues of process development. These extra functions may be addressed by changes to the vector genetic material, that is, by including cell-specific promoters to avoid off-target effects or by modification of the physical shell of the vector, for example, a liposome formulation or indeed retro/lentiviral (R/LV) particles. The latter will be the topic of this paper. R/LV vectors are complex structures, which are inherently difficult to analyse in detail, as a result of their biological origin. While this inherent biocompatibility is at least partially responsible for their success, nevertheless a more controlled environment at the surface of R/LV particles would be beneficial for both, simplifying the regulatory/manufacturing aspects of gene therapy approaches as well as enhancing efficacy and safety of such approaches. More specifically, possibilities may be found in several areas: (i) easy concentration and purification of vector stocks for clinical use, (ii) being able to monitor administration and distribution of vectors (both of which can be achieved by suitable labeling), (iii) navigating the host response, especially a patient's immune response, and (iv) targeting of vectors to specific organs, tissues, or cell types (for a more extensive discussion see [1] and see also Figure 1 for an overview). 

Ultimately, vectors for gene therapy need to meet the requirements agreed upon by manufacturers, regulatory agencies, and clinicians to satisfy the potential demand. For the production of gene therapy vectors, reproducible and safe methods for purification and concentration of cell culture supernatants containing the recombinant viral particles are needed. Ideally they should be easily upscalable later. However, challenges arising in that field are difficult to foresee. To date, methods depend on physical properties of viral vectors, such as size, charge or biological surface properties which may vary from one production system to the next [2, 3]. Presence of a tag on the surface of the vector compatible with large-scale purification/concentration methods [2, 3] could be beneficial, especially if broadly distributed amongst available vector species. In the case of *in vivo* delivery of gene therapy vectors, it is mandatory to be able to follow the physical presence of vector particles in the patient, to be able to assess efficacy of administration and delivery. This cannot be achieved by genetic transduction markers (i.e., reporter genes), a tool commonly used in research settings. On the one hand introduction of such markers within the framework of an R/LV for clinical use will be problematic from the regulatory and safety perspectives and on the other hand these markers define transduction of cells only and not the distribution of the physical vector shell within the patient. As a consequence, the degree and character of vector loss before a target cell reached cannot be assessed in such a manner. However, this information is crucial for evaluating the success of any gene therapy approach [4]. Therefore, a broadly distributed marker, easily detectable with high specificity and sensitivity as well as low immunogenicity and a stable enough linkage to the vector to prevent label leakage would be ideal. In the case of *in vivo* delivery the complexity of the entire organism must be navigated, with one of the primary concerns being the immune system which may contribute significantly to vector loss before the target has been reached. Manipulating vector surface to minimise immunogenicity seems an adequate measure to counteract unwanted immune responses. This can be achieved by introducing factors regulating immune responses, such as CD59 [5]. Conversely, stimulation of immune reactivity may be beneficial, to introduce supplementary therapeutic effects (i.e., stimulation of tumour targeted immune reactions). Finally, infection targeting has been an alluring research goal in the field of gene therapy despite its remaining elusive thus far. How to make sure that only a specific subset of cells is infected, thus providing increased safety and efficacy? Since molecules involved in finding and entering target cells are located at the surface of R/LV vectors, manipulation of this compartment is vital for defining novel targeting properties of the vectors. A range of different approaches have been tried with limited success [6]. Subsequently, additional strategies are called for.

## 2. *Postexit* Surface Engineering: Why Modify Virus Particles after Their Exit from the Cell?

The manipulations to modify R/LV vector surfaces can be undertaken before or after the virus has left the producing cell, that is, before or after exit. What are the advantages of manipulating viral particles as compared to changing the virus producing cells genetically? *Preexit* strategies are based on genetic manipulation of virus vector producing cells by transfection- or transduction-based methods. Factors that can be produced are either nucleic acids or proteins, since such *preexit* modifications are inherently biological processes. Display of a small molecule compound is more difficult in these circumstances. In contrast, *postexit* modifications can be more (bio)chemical in nature, making small molecules a more accessible target. Whereas most *postexit* strategies may also be applied *before exit* (directly on producing cells' membranes, thus managing incorporation of the modification in the budding virus), the opposite is not the case. Some advantages common to *postexit* methods are their flexibility, speed, comparable ease of use, and somewhat more controlled/reproducible modification conditions and outcomes. If a virus is difficult to culture, or the knowledge about its genetics and/or molecular biology is scarce, *preexit* strategies may not be applicable. *Postexit* methods are independent of the genetic setup of the R/LV vectors; thus, can be employed on a wider range of vectors and do not need to be established newly but only optimised for each new therapeutic/marker gene. If targeting or modulation of the immune system is the objective, modifying molecules may be changed with relative ease, also potentially opening new routes to personalised, custom-made therapies. Since no nucleic acid production or protein expression is required during the actual vector modification, procedure times can be kept brief and modifying molecules are not limited to factors naturally produced by cells. Stoichiometry rules usually apply and allow for the controlled deposition of varying amounts of modifying agents. On a more practical note, *postexit* procedures may be implemented on top of existing production lines, rather than having to design processes completely new. Also the disadvantages of *postexit* strategies need to be considered: while the actual modification may be kept simple, preparation of compounds for the procedure may be quite complex. Additionally, *postexit* steps inherently reduce maximum infectivity, if only due to the time it takes to introduce the modification [7]. As a consequence, *post-exit* modifications need to be quick to be useful and may be initiated as early as possible, potentially already during production of vectors. After the process it is important to separate viruses from nonassociated modifying molecules, since they may interfere with downstream processes, thus adding an extra step in the production or manufacturing process. This is an important feature of any *postexit* procedure, which is often underestimated, and subsequently needs to be addressed thoroughly. *Postexit* procedures may be inherently reversible; thus the loss of modification over time needs to be analysed critically. Additionally, issues regarding regulatory affairs connected with *postexit* procedures are unclear. Summing this up, *postexit* strategies may be preferable if a wider range of modifications need to be implemented on the same viral vector, that is, to ensure infection targeting of different cell types or when a common modification has to be delivered to a wider range of viral recipients, such as in the case of tagging for purification or monitoring applications.

## 3. Methodology: How Can R/LV Surfaces Be Modified after Their Exit from Cells?

Generally modification of R/LV vector surfaces is achieved by genetic manipulation of the virus producing cell lines. The most often used method is pseudotyping with proteins of heterologous viral origin [1, 35] or the use of fusion proteins consisting of mixed viral/nonviral sequences [1, 36, 37]. These require the activity of the cellular expression machinery [1]. In contrast, *postexit* procedures fall roughly into three categories: (i) direct covalent modification, (ii) the use of membrane-topic moieties, and (iii) the use of adaptor systems (see Figure 1 and Table 1 for an overview). Mixed forms are possible, that is, the delivery of an adaptor site by transfection or covalent chemical modification.

(i) Direct covalent modification. Theoretically, the most straightforward approach is the covalent modification of structures on the virus surface by means of a directed, controlled chemical reaction that targets more or less specific compounds on the virus surface. Due to the inherent increased chemical stability of naked virus, this strategy is more often used on adenoviruses and adeno-associated viruses; that is, the successful covalent association of polymers and polypeptides has been achieved [38, 39] as well as radiolabeling by iodination [40]. Conversely, attempts to direct covalent modification on enveloped virus particle have been rare. However, radiolabeling by iodination has been achieved on enveloped viruses as early as 1975 [8]. The effect of this modification on viral infectivity is not well documented. A successful example for covalent manipulation is the attachment of monomethoxy-poly(ethylene)glycol (PEG) to LV vectors [10]. In this case an activated form of PEG is covalently attached to lysine residues on proteins displayed on the virus. PEGylation reduces the susceptibility of these vectors for an attack by the complement system, while not disturbing transduction [10]. This constitutes a manipulation of the host's immune system. In another early attempt, Moloney murine leukemia virus (MoMLV) was modified by chemical addition of lactose moieties in order to change viral tropism [9]. Introduction of these residues was supposed to specifically infect hepatocytes expressing receptors recognizing the carbohydrate moieties on the viral vectors. However, the modification resulted in severely reduced infectivity of R/LV particles. Direct chemical biotinylation of retroviral vectors has also been demonstrated, using sulfo-N-hydroxysuccinimide-biotin on MoMLV derived vectors [11]. Neutravidin was covalently linked to polylysine. The resulting compound was then associated to the biotinylated vector. The aim of the study was to allow transduction of human cells with ecotropic MLV vectors, which normally cannot infect human cells. In this case, progeny of modified viruses would lack the modification; hence infection of adjacent cells would not occur, even if replication competent vectors were generated. This would contribute to the safety of gene therapy approaches. More recently, developments in bio-orthogonal chemistry could bring new impetus to the field. Bio-orthogonal chemistry describes the possibility to allow controlled, specific chemical reactions amidst the background of a biological system, that is, in cell culture. For example, cell surfaces can be modified by oxidation of sialic acids present on glycosylated surface proteins by periodate, generating reactive groups, which in turn can be modified by conjugation of aminooxy-functionalised compounds [41]. When this technique was applied to cells producing VSV-G pseudotyped MoMLV, resulting viral particles carried the modification [13]. They used this to introduce aminooxy-biotin and could subsequently associate magnetic particles to the virus, facilitating purification and concentration of virus preparations. This approach appears to also be applicable to viral particles after exit [13]. Biological chemistry, by developing bio-orthogonal methods, appears to have great potential for novel types of modification. The loss of modification will be a minor issue, due to the covalent nature of the association. However, in most cases direct protein modification is difficult, since chemical procedures may interfere with protein stability and/or function. In this regard, “softer” methods are called for.

(ii) Membrane-topic compounds. R/LV vector particles are covered with a lipid bilayer membrane, the envelope, defining the outer surface of the virus. Introducing molecules with an affinity or tropism for lipid structures is another strategy to modify enveloped viral vectors, at least semi-specifically. When using membrane-topic compounds, usually no preparatory “activation” of membranes would be necessary, as it is commonly required for direct chemical modifications, and also in cases when membrane-bound adaptors are used. This may provide increased biocompatibility, compared to other strategies. However, off-rates, that is, loss of modification need to be monitored. To date, two compounds have been used to achieve viral surface engineering, chemically synthesized function-spacer-lipid (FSL) constructs and recombinant *in vitro* produced and purified glycosylphosphatidylinositol-(GPI-) anchored proteins. Additionally, other classes of compounds could be suggested for virus modification: artificial GPI-mimetic moieties [17] and membrane-topic constructs combining peptide spacers and myristoylation [19] as well as oleyl chains linked to PEG [18]. Modification of herpes virus particles with envelope penetrating lipophilic radioactive labels has been demonstrated and was used for biodistribution studies [16, 42]. The property of Indium-111 8 hydroxyquinoline (^111^-In oxine) complexes to traverse eukaryotic membranes was exploited to radioactively label herpes simplex virus type 1 (HSV-1) and demonstrate its biodistribution in rats. Finally, lipophilic tracer dyes such as the long-chain dialkylcarbocyanines, in particular DiI, are another group of membrane-targeting agents [43] which may be used to modify viral particles [44].

Generally, chemically synthesised compounds need to meet three requirements to be able to be useful for modification of membrane structures: firstly a strong hydrophilic signal to mediate association to the membrane, secondly a reactive site that allows linkage to the functional moiety of the construct, and finally, the stability and functionality of both the functional part and the association need to be assured, often requiring additional chemical structures, located in the linker portions of molecules. Function-spacer-lipid (FSL) constructs have been used to modify cells *in vitro* and *in vivo *[43, 45–48], as well as enveloped viral particles [14]. To date small fluorescent molecules [14], biotin [45, 46] and short peptide [47] sequences, but no complete proteins, have been used for modification. Vesicular stomatitis virus (VSV), measles virus (MV), and influenza virus (IV) have been modified with fluorescein- or radiolabeled FSL constructs [14]. Fluorescent labels were used to demonstrate *in vitro* attachment to cells and radiolabeled constructs were used to show biodistribution in a mouse model. Although modification of R/LV vectors was not demonstrated using FSL constructs yet, it seems likely that this can be achieved. 

GPI-anchoring is a form of posttranslational modification occurring in all eukaryotic cells [49]. Proteins marked for GPI-anchoring contain a GPI signaling sequence (GSS) at the C-terminal end. The GSS is recognized in the endoplasmic reticulum by the transamidase enzyme complex where it is cleaved and replaced by the preformed GPI-anchor. GPI anchored proteins have a variety of different functions, from complement regulatory activity (CD55 and CD59) and restriction of viral transmission (Tetherin) [50] to signal transduction (Thy1) [51]. GPI-linked proteins are targeted to the outer surface of the cell membrane [52, 53] and are frequently associated with membrane microdomains or lipid rafts (LR) [54]. There is evidence to suggest that LR are the site of viral assembly for certain enveloped viruses, for example, HIV-1 [55, 56]. Additionally, processes that release GPI-linked proteins into the medium with intact GPI anchors are reversible and it has been shown in a variety of *in vitro* and *in vivo* systems that GPI-linked proteins can be reinserted into cell membranes [57–62]. This hypermobility extends to the reintegration of purified GPI-anchored proteins to lipid membranes of cells [54, 63] and viruses [7, 15] for technical purposes. This process has been termed cellular or viral painting, respectively, or, when applied generally to all lipid membranes, molecular painting. This was first described for the GPI-linked model protein CD59his which associates to viral vectors based on MLV and HIV-1 [7]. The association is dependent on the presence of the lipophilic parts of the GPI anchor [15] and painted virus particles remain infectious after insertion of the GPI-linked protein, albeit at reduced efficiencies caused by the duration of the painting process, rather than the actual introduction of GPI-anchored molecules into the viral outer shell [7]. Molecular painting of retroviral vectors with CD59his leads to an increased resistance to complement activity (unpublished data). In addition to CD59, different forms of green fluorescent protein have been used for painting, indicating that the use of monomeric proteins is required for viral painting. GPI-anchored proteins can be attached to a range of enveloped viral particles other than R/LV, that is, herpes virus and influenza virus [15] without repeated genetic manipulations of the virus producing cells. Additionally, deposition of two GPI-anchored proteins simultaneously is possible, subsequently enabling the introduction of multiple functionalities in one go. Introducing a GPI anchor to any given protein is achieved in the same way fusion proteins are made: following genetic engineering, the recombinant protein is translated and the amino acid sequence describing the GSS is included to the nascent polypeptide, and thus artificially GPI-anchored proteins are produced. A range of recombinant GPI-anchored proteins have been produced including GPI-anchored green fluorescent protein (GFP), interleukin 2 (IL2), epidermal growth factor (EGF), and the main HIV receptor CD4 [64] and are currently being investigated for their molecular painting potential. Molecular painting of viruses may be the method of choice for *postexit* modification of enveloped viral particles with complex protein functions in situations where a degree of flexibility is preferred. 

(iii) Adaptors. The third approach uses mediator or adaptor molecules. Such adaptors may either be soluble, for example, bispecific bridging components [25] or membrane-bound, for example, by introducing a biotin acceptor peptide (BAP) into a membrane-bound protein [21]. Soluble adaptors may either carry the desired function directly or require linkage to another element. Soluble adaptors need to bind their receptors strongly, to keep leakage rates low. Mainly, adaptor molecules have been used to enable targeting strategies in gene therapy. In these cases bispecific molecules or assemblies were used, contacting one molecule present on the virus and another on the cells which are targeted for infection. Such bispecific adaptors or bridge complexes can take different forms, for example, two different antibodies, modified with biotin, can be linked via avidin or streptavidin thus ensuring specific binding to viral surface proteins and the target molecule on the cell at the same time [20]. Such a system has been proposed as early as 1989, showing directed infection of MHC class I and II expressing cells with murine retroviruses [20]. This is highly flexible and versatile, since a wide range of antibodies which can be biotinylated are available. Pretreatment of the viral vector with the antiviral antibody would effectively neutralize the viral particle, thus increasing the safety of the application. However, the streptavidin/biotinylated antibodies complex constitutes quite a bulky molecule, which may interfere with viral dissemination or entry. Alternatively, a chimeric protein, in which the binding partner for the viral attachment protein is coupled to a ligand, binding to a target molecule on the cell surface may be used. Vectors pseudotyped with avian sarcoma and leukosis virus (ASLV) have been employed in such strategies. The chimeric bridge protein consisted of the extracellular domains of the cellular receptor for ASLV, fused to ligands such as epidermal growth factor (EGF), vascular endothelial growth factor (VEGF), or heregulin, thus targeting cells expressing the respective receptors [25–27]. Since these receptors are commonly overexpressed on tumor cells, the approach is already of some medical relevance. Instead of the ligands, also single-chain antibodies may be used. An approach has been used for targeting cells expressing a tumor-specific form of the EGF receptor [26]. A similar system has been used recently, to retarget HSV-1 vectors to tumor cells carrying the carcinoembryonic antigen (CEA) [28]. Taking this approach one step further, bispecific antibodies may be used as bridging elements. So far this has been tried on adenoviral vectors to retarget or increase immunogenicity [29, 30]. When using membrane associated adaptors, in most cases, avidin or streptavidin molecules engineered to contain a transmembrane domain are utilised, due to their extraordinarily strong affinity to biotin and the comparative ease with which biotin can be attached to a wide range of compounds from DNA to antibodies. Avidin and streptavidin molecules are available in a wide range of modifications, tailor-made for different applications [65]. The main advantage of this system is its flexibility, since factors attached to avidin or streptavidin can be exchanged. Such a system has been implemented by fusing avidin and streptavidin with the transmembrane domain of VSV-G [4]. The binding of biotin to such vectors was demonstrated and they could be used for dual imaging and for targeting applications. Other approaches lead to the biotinylation of the lentiviral vector. This can be achieved by direct chemical modification [11] or after addition of a biotin-adaptor peptide (BAP), a site for specific enzymatic biotin ligation [22, 23]. The bacterial enzyme, biotin ligase, has to be provided as a form of metabolic engineering to allow the modification of the BAP-containing protein. Both a cellular protein, low-affinity nerve growth factor [22], and a viral protein, Sindbis virus glycoprotein [21], have been modified in such a way to generate novel LV vectors. The latter mixes four different strategies to modify R/LV viral vectors, two of which may be carried out after exit: pseudotyping of an LV vector with a chimeric envelope molecule, containing an adaptor element, added by enzyme-mediated covalent chemical modification. Alternatively, membrane proteins binding antibodies may be used to modify viral surfaces. Insertion of immunoglobulin G-binding domains (the ZZ domain of staphylococcal protein A) into the Env protein of MLV vectors allowed for the binding of specific antibodies directed against the EGF receptor HER2. However, infectivity was significantly reduced, as it would be expected [31]. A similar approach utilizes a fusion of the same antibody binding domain with Sindbis envelope glycoproteins [32]. Another adaptor approach for modification or enveloped viral vectors may be designed around the use of split inteins [34]. Comparably to *trans*-splicing of pre-mRNAs, protein elements derived from two different proteins may be fused together after exit in a covalent way. When one of the two elements to be joined is placed in the membrane of cells or viral particles, the reaction may be used to link different functionalities to membranes. The peptide sequences containing the intein are effectively removed during the process. This approach has been used for the fluorescence modification of cells *in vitro *[34], but not yet for modification of viral vectors. Finally, by feeding virus producing cells on modified amino acids or carbohydrates, adaptor sites for covalent chemical modifications by “click” chemistry can be introduced to viral vectors, an approach that has been used for the modification of adenoviruses [33]. The main disadvantage of adaptor systems is that an additional, separate element is necessary for the system to work, thus introducing a new level of complexity. Additionally, adaptors may dissociate from one or the other binding partner and competition from serum antibodies for binding *in vivo* may significantly enhance dissociation [66].

## 4. Summary

Successful delivery of therapeutic nucleic acids in clinical practice will depend on a variety of factors, from efficient production and purification of stocks to immune evasion and infection targeting. Modification of R/LV vector surfaces can contribute to amendments of viral vector preparations in these aspects. Under certain conditions, that is, when a high degree of flexibility is asked for, these changes may be preferably carried out after the viral vector has left the producing cell (after exit). A range of different techniques, described in this paper, have been used to achieve such *postexit* modifications in a research setting, mostly via direct chemical modifications, via membrane-topic compounds, or via various adaptor systems, but may be applicable also for clinical purposes.

## Figures and Tables

**Table 1:** Approaches to post-exit modification of R/LV vector surfaces.

Type	Virus/vector	Modification	Objective	References
Covalent chemical				
Iodination	(AV), VSV, **RV**	Radionuclides	L	[8]
Reductive amination	**RV**	Lactose	R	[9]
Conjugation	**LV**	Poly (ethylene) glycol	H	[10]
Biotinylation	**RV**, HV	Biotin, different secondary	R	[11, 12]
PAL chemistry*	**RV**	Biotin, Alexa fluorophore	P, L	[13]
Membrane-topic				
FSL	VSV, MV, IV	Fluorescein, Biotin	L	[14]
Molecular painting	**RV**, **LV**, HV, IV	CD59, GFP	L, H	[7, 15]
Membrane-traversing**	HV	Radionuclides	L	[16]
Synthetic GPI**	n.a.	n.a.	n.a.	[17]
Oleyl/PEG**	n.a.	Streptavidin, GFP, mAB	L	[18]
Myristyl/peptide**	n.a.	CD59	(H)	[19]
Adaptor-based				
(Strept)avidin (soluble)	**RV**	Streptavidin, MHC	R	[20]
(Strept)avidin (membr.)***	**LV**, BV	Biotinylated radionuclids, antibodies and ligands	L, R	[4]
Biotinylation	See above			
Biotin acceptor peptide***	**LV**, BV	Biotin, different secondary	P, R	[21–24]
Bridging molecules	**RV**, HV	Heregulin EGF, VEGF AntiCEA	R	[25–28]
Bispecific antibodies**	(AV)	AntiCD40 AntiEndoglin	R, H	[29, 30]
Antibody binding (membr.)***	**RV**	AntiHER2 AntiP-GP	R	[31, 32]
“Clickable” Adaptors**	(AV)	TAMRA	(L)	[33]
Split inteins^∗∗/∗∗∗^	n.a.	GFP	(L)	[34]

*Mostly pre-exit, **not tried on R/LV, ***requires genetic modifications (transfection/transduction) to deliver part of the system. RV: retrovirus, LV: lentivirus, VSV: vesicular stomatitis virus, MV: measles virus, IV: influenza virus, HV: herpes virus, BV: baculovirus, AV: adenovirus. Lenti/retroviruses are in bold and underlined, viruses in brackets are naked viruses. P: preparation, L: labeling, H: host responses, R: range of infectivity. Objectives in brackets have not been carried out on enveloped viruses. CD59 protectin, GFP green fluorescent protein, MHC major histocompatibility complex, EGF epidermal growth factor, VEGF vascular endothelial growth factor, CEA carcinoembryonic antigen, HER human epidermal growth factor receptor 2, P-GP permeability glycoprotein, TAMRA Carboxytetramethylrhodamine.
